# A Comprehensive Survey of Vision-Based Human Action Recognition Methods

**DOI:** 10.3390/s19051005

**Published:** 2019-02-27

**Authors:** Hong-Bo Zhang, Yi-Xiang Zhang, Bineng Zhong, Qing Lei, Lijie Yang, Ji-Xiang Du, Duan-Sheng Chen

**Affiliations:** 1 Department of Computer Science and Technology, Huaqiao University, Xiamen 361000, China; 17013083014@hqu.edu.cn (Y.-X.Z.); bnzhong@hqu.edu.cn (B.Z.); leiqing@hqu.edu.cn (Q.L.); yanglijie@hqu.edu.cn (L.Y.); dschen@hqu.edu.cn (D.-S.C.); 2 Xiamen Key Laboratory of Computer Vision and Pattern Recognition, Huaqiao University, Xiamen 361000, China

**Keywords:** action detection, action feature, human action recognition, human–object interaction recognition, systematic survey

## Abstract

Although widely used in many applications, accurate and efficient human action recognition remains a challenging area of research in the field of computer vision. Most recent surveys have focused on narrow problems such as human action recognition methods using depth data, 3D-skeleton data, still image data, spatiotemporal interest point-based methods, and human walking motion recognition. However, there has been no systematic survey of human action recognition. To this end, we present a thorough review of human action recognition methods and provide a comprehensive overview of recent approaches in human action recognition research, including progress in hand-designed action features in RGB and depth data, current deep learning-based action feature representation methods, advances in human–object interaction recognition methods, and the current prominent research topic of action detection methods. Finally, we present several analysis recommendations for researchers. This survey paper provides an essential reference for those interested in further research on human action recognition.

## 1. Introduction

Human action recognition has a wide range of applications, such as intelligent video surveillance and environmental home monitoring [1,2], video storage and retrieval [3,4], intelligent human–machine interfaces [5,6], and identity recognition [7]. Human action recognition covers many research topics in computer vision, including human detection in video, human pose estimation, human tracking, and analysis and understanding of time series data. It is also a challenging problem in the field of computer vision and machine learning. At present, there are many key problems in human action recognition that remain unsolved.

The key to good human action recognition is robust human action modeling and feature representation. Feature representation and selection is a classic problem in computer vision and machine learning [8]. Unlike feature representation in an image space, the feature representation of human action in video not only describes the appearance of the human(s) in the image space, but must also extract changes in appearance and pose. The problem of feature representation is extended from two-dimensional space to three-dimensional space-time. In recent years, many kinds of action representation methods have been proposed, including local and global features based on temporal and spatial changes [9,10,11], trajectory features based on key point tracking [12,13], motion changes based on depth information [14,15,16], and action features based on human pose changes [17,18]. With the successful application of deep learning to image classification and object detection, many researchers have also applied deep learning to human action recognition. This enables action features to be automatically learned from video data [19,20]. Additionally, some studies have reviewed these action recognition approaches. However, these review articles have only discussed specific aspects, for example, spatial temporal interesting point (STIP)-based human action recognition methods, human walking analysis approaches, and deep learning based methods. Many new methods have recently been developed, particularly regarding the application of depth learning methods to feature learning. Therefore, a thorough survey of these new human action recognition approaches is of considerable interest.

In this work, we summarize many recent works and present a new survey of research on human action recognition techniques including action classification (focusing particularly on the action representation methods of action classification), human–object interaction recognition, and action detection methods. The action classification methods are summarized into methods based on handcrafted features and those based on feature learning. These methods can be applied to different types of data.

From a data perspective, most reviews of human action recognition are limited to approaches based on specific data, such as RGB data, depth data, or skeleton data. Most of the above-mentioned works focus on human action recognition in RGB video data. With the development of depth cameras, depth data are now widely used in many computer vision tasks, especially the estimation of human poses to extract human skeleton data. Moreover, the latest research results on human detection and pose estimation [21,22,23] in RGB video show that deep learning-based methods can achieve efficient and accurate multihuman pose estimation in complex scenes. Several human action recognition techniques based on depth sequences and skeleton data have also been proposed [5,17,24]. To a certain extent, these methods have solved some of the problems of human action recognition with RGB cameras or video, and have demonstrated good recognition performance. Works on human action recognition methods using depth and skeleton data have been reviewed by Ye et al. [15], who summarized human action recognition methods using depth data and Presti et al. [6], who discussed human action recognition methods based on 3D skeleton data. In addition, Guo et al. [25] analyzed human action recognition methods from still images. Although some of these review articles cover data fusion approaches, they still provide a narrow summary of human action recognition approaches toward specific types of data. To solve this problem, in this work, we summarize human action recognition methods applicable to different types of data and involving handcrafted feature-based and feature learning methods.

Another important aspect of the status on human action recognition research is that most studies concentrated on human action feature representations. The processed image sequence data are usually well-segmented and contain only one action event. This then becomes a classification problem. However, there are two key problems in the actual scenario: interaction recognition and action detection. Interaction refers to actions that involve more than two people or actions between people and objects, such as carrying a knife or playing an instrument. Action detection refers to locating the position at which an action occurs in time and space from image sequence data that have not been segmented. In recent years, interaction [26,27,28] and human action detection [29,30,31] have become prominent research topics. However, there is no overarching summary of the methods applicable to these two issues. To this end, the state of research on interaction recognition and action detection is reviewed in this paper.

Human action recognition surveys similar to our work have been presented in recent years [32,33,34,35,36]. For example, Vishwakarma et al. [34] provided a comprehensive survey of human action recognition methods developed from 2008 to 2012. Those human action recognition methods were divided into three different levels: human detection (low-level vision), human tracking (intermediate-level vision), and behavior understanding methods (high-level vision). Similarly, Subetha et al. [33] used the same strategy to summarize action recognition methods from 2013 to 2016. Wang et al. [35] summarized existing literature in the context of three aspects: sensor modality, deep models, and application. However, in contrast to those works, we here divide human action recognition into action classification and action detection. Furthermore, both handcrafted feature-based methods and deep learning-based methods are discussed in this work. The study most similar to the present work is that by Herath et al. [32], which also summarizes action recognition methods according to handcrafted feature-based methods and deep learning-based methods. However, in our work, these two methods are further discussed with consideration of different data types. Herath et al. [32] discussed the classification methods of human action only, omitting a survey of interaction recognition and action detection methods. Note also that Yu et al. [36] performed a recent survey of literature on action recognition and action prediction; the latter predicts the future state of human actions based upon incomplete action executions.

**Figure 1:**
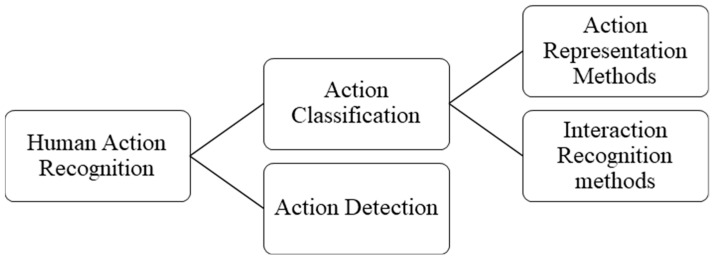
Classification framework for human action recognition methods.

To collocate together previous surveys and fill the gaps between them, we here provide a comprehensive survey of human action recognition techniques (see Figure 1), including action feature representation methods, interaction recognition methods, and action detection methods. To perform this survey, various studies are reviewed in our work. When preparing this paper, a key problem was literature selection. First, a survey framework was implemented to span the main effective methods of human action recognition. The selected works were then collected based on relevant keywords, such as “action recognition”, “action feature representation”, “interaction recognition”, and “action detection”. Second, based on the initially selected literature, relevant works were added. Finally, based on the action recognition dataset, further studies were added to complete this work. In our study, most literature was collected from academic journals and conferences on computer vision. The articles published in important journals and conferences such as IEEE Transactions on Pattern Analysis and Machine Intelligence (TPAMI), IEEE Transactions on Image Processing (TIP), International Conference on Computer Vision and Pattern Recognition (CVPR), IEEE International Conference on Computer Vision (ICCV), among others, will be preferred. Subsequently, we expand upon these articles to gather more relevant work. The acceptance criteria of the literature are high citations or good recognition performance. Simultaneously, to make our work cover more different methods, for similar methods, we only choose a basic or representative method for detailed discussion.

The contributions of this paper are threefold:

(1) We provide a comprehensive survey of human action feature representation methods involving different data types, including hand-designed action features and deep learning-based action feature representation methods for RGB, depth, and skeleton data.
(2) Different from most previous reviews, which discussed methods of action modeling and action classification only, human–object interaction recognition and action detection methods are also summarized in this work.
(3) We propound some suggestions for future research on human action recognition.

The remainder of this paper is organized as follows. An overview of human action recognition is provided in Section 2. The human action feature presentation methods are then introduced in Section 3, including handcrafted features based on RGB video, handcrafted features based on depth and skeleton data, and deep learning-based methods. In Section 4, we introduce approaches that recognize human–object interactions. In Section 5, methods of human action detection are summarized. In each section, we present an overview of recent developments and the classical methods of human action recognition. Finally, some research suggestions are provided in Section 6.

## 2. Overview of Human Action Recognition

From the perspective of data type, research on human action recognition can be divided into methods based on color (RGB) data [9,37,38,39,40] and methods combining color and depth data (RGBD) [14,17,24,41,42]. The human action recognition approaches for these data, following the progress of machine learning research, can be categorized as either hand-designed features with machine learning methods or end-to-end deep learning algorithms. Regardless of data type and computing method, the core aim is to extract robust human action features. Many action features have been proposed for RGB data, such as spatiotemporal volume-based features [10,43], spatiotemporal interesting point features [9,44], and joint trajectory features [12,13]. However, factors such as camera movement, occlusion, complex scenes, and the limitations of human detection and pose estimation methods limit the performance of human action representation and recognition based on handcrafted features.

Because depth data are stable with respect to changes in environment and background and allow objects to be quickly segmented according to depth, the application of depth sensors enables real-time, robust human pose estimation. Human action recognition methods based on depth information and skeleton sequences demonstrate high recognition accuracy and low time complexity. These methods are popular in human action recognition research [16,17,18,24,41]. However, the accuracy and cost of depth sensors mean that depth- and skeleton-based action recognition methods are currently only applicable over limited ranges and in specific environments. There are three types of commonly used depth cameras: triangulation (with two camera views), time-of-flight (TOF) cameras, and structured-light-based cameras. Structured-light and TOF-based depth sensors are easily affected by light, with large errors and low precision in outdoor environments. The cost of the two-camera system is lower, but the depth information calculation has higher complexity and cannot be applied in darker environments. In addition, there are other sensors that can be used to measure depth, such as laser scanners; however, these devices are expensive and unsuitable for video surveillance and home monitoring.

Unlike handcrafted action features, deep learning methods perform well with regard to automatic feature learning from images. This provides a new insight into human action recognition, and many researchers have attempted to use deep learning methods to extract action features from RGB, depth, and skeleton data. Such data are applicable to multimodal feature learning [19,20,42,45] from deep networks, such as appearance/image information, optical flow sequences, depth sequences, and skeleton sequences. Deep learning networks can learn human action features from single-mode data or multimodal fusion data. As the appearance sequence and optical flow sequence are relatively easy to obtain, most deep learning methods adopt the appearance sequence and optical flow sequence as their input, with few depth- and skeleton-based techniques. However, recent high-efficiency multiperson pose estimation methods based on deep learning [21,22,23] have drawn increased attention to human action feature learning based on skeleton sequences, and this is now a prominent research topic in the field of human action recognition [46].

Human action recognition can be divided into action classification and action detection. Action classification is the analysis of a segmented video containing only a single action that must be classified into a defined action category. Action detection detects the start and end times of each action in the video, locates their position in space, and identifies the action category. In early research, the study of human action recognition focused on the action classification problem. With the development of related research topics, such as machine learning, object detection, and human pose estimation, research on the more challenging human action detection has become popular in recent years [47,48,49,50,51].

Many studies have considered human action recognition. According to the complexity of the human action, previous techniques can be categorized into the following four classes of action semantics [1] from low to high: primitive action recognition [52], single-person action recognition [6,9], interaction recognition [26], and group action recognition [53]. Primitive action refers to basic movement changes of human body parts, which is the minimum structure of action. These changes can be combined into more complex actions through various elementary movements. For example, “waving”, “lifting a foot”, and “bending” are common primitive movements, and gesture recognition is a common primitive action recognition problem. Single-person actions refer to the set of primitive actions of a single person, such as “walking”, “punching”, and “jumping”. Interaction refers to actions involving the relationship and movement between humans and objects, such as “carrying a knife” and “playing an instrument”. Group actions refer to those that occur in a group composed of many people or objects, such as a “parade”, “group meeting”, and “group fighting”. Previous research has mainly focused on the first two levels of action recognition. Although interaction recognition has received more attention in recent years, the research on group action recognition is still in its infancy.

In addition, in current research, only action classification problems span these four different semantic levels of action. Previous research has focused on the first two levels of action classification. Although interaction recognition has received greater attention in recent years, research on group action recognition remains in its infancy. Meanwhile, the key problem regarding classification of primitive action and single-person action is action feature representation. However, research on action detection mainly focuses on primitive action and single-person action. Therefore, to render the survey presented in this paper more pertinent, we summarize and analyze action recognition methods from three aspects: feature representation, interaction recognition, and action detection for primary and single-person actions, as shown in Figure 1.

The following sections present a comprehensive survey of human action recognition methods, including human action feature extraction methods, interaction recognition methods, and action detection methods.

## 3. Human Action Feature Representation Methods

### 3.1. Overview of Handcrafted Action Features for RGB Data

Handcrafted features are intended to capture the human movements and spatial and temporal changes in video representing action, including spatiotemporal volume-based action representation methods, STIP-based methods, action representation methods based on the trajectory of skeleton joints, and action representation based on human image sequences. These features are mainly used in classic machine learning methods such as Boost, support vector machines, and probability map models to recognize action.

The spatiotemporal volume-based methods are template matching techniques, but unlike object recognition in image processing, they use a three-dimensional spatiotemporal template for action recognition. The key to these methods is to construct a reasonable action template and perform effective matching based on this template. This was the earliest approach to human action recognition, with the main methods being those developed by Bobick and Davis et al. [54], who proposed the motion energy image (MEI) and motion history image (MHI) for representing action; Zhang et al. [55], who used polar coordinates to divide the central region of the human body in MHI and a Motion Context descriptor based on SIFT to represent the action; Klaser et al. [56], who extended the histogram of gradients (HOG) feature of an image to the space-time dimension and proposed the 3DHOG feature to describe human actions in video; and Somasundaram et al. [57], who used sparse representation and dictionary learning methods to calculate the self-similarity of a video in the temporal and spatial dimensions, thus providing a representation of human actions.

When the camera is fixed, these methods can use background subtraction techniques to obtain shape information such as human silhouettes and contours. However, in the case of complex scenes and camera movements, accurate silhouettes and contours are difficult to obtain, and in cases where the human body is occluded, an accurate human appearance cannot be identified. To determine multiple actions in the same scene, most methods use a sliding window, but this approach is computationally expensive.

STIP-based methods, which are widely used for action recognition, extract the key region of movement change from a video to represent action. Similar to the local features for object recognition in images, STIP methods must determine which key region detection method to use, which feature vector to use to describe the key region, and which classification algorithm to employ. Therefore, most of these methods are extended from object recognition methods applied to images. The “interesting points” in STIP generally refer to the position that changes most dramatically in the spatiotemporal dimension [9]. Popular STIP methods include 3D-Harris spatiotemporal feature points [58] and similar or improved techniques [59]. The main idea is to extend the local feature detection technology from images to the 3D spatiotemporal domain. The feature descriptors are then computed and the visual dictionary for representing human actions is learnt. Nguyen et al. [10] proposed a key region extraction method based on a spatiotemporal attention mechanism to construct a visual dictionary and action features. Peng et al. [60] reviewed and contrasted methods based on local spatiotemporal features and visual dictionary construction, and proposed a hybrid supervector human action representation method that achieved good performance with several common human action datasets. Nazir et al. [61] integrated the 3D-Harris spatiotemporal features and 3D scale-invariance feature transform detection methods to extract the key regions of a video, and used traditional visual word histograms to represent human actions.

Methods based on spatiotemporal features have attracted the attention of many researchers. The main advantage is that such methods do not require preprocessing such as background segmentation or human detection. Local features offer scale and rotation invariance, are stable under illumination changes, and are more robust to occlusion than other methods. However, the spatiotemporal feature points are easily affected by changes of camera view. In the case of background motion and camera motion, methods based on local spatiotemporal features will generate many background feature points, and the motion estimation of the object will suffer from large errors.

Trajectory-based features use the tracking path of key points or the joints in the human skeleton to represent actions. The representative trajectory-based action recognition method is the improved dense trajectories (iDT) proposed by Wang et al. [13]. Sampling dense point clouds from a video frame, the displacement information is calculated by tracking these feature points using an optical flow approach. The background motion can be eliminated, allowing the motion trajectory to be calculated so that the feature is more focused on the description of the human motion. A number of studies have improved iDT or incorporated its features. Gaidon et al. [62] used split clustering to analyze local motion trajectories, and used the clustering results to represent different motion levels in calculating human action features. Wang et al. [63] fused the results of human detection into iDT features, thereby reducing the interference of background trajectories. Peng et al. [64] used stacked Fisher vectors to represent human actions based on iDT features, resulting in one of the more successful methods for improving iDT. The main advantage of trajectory-based action recognition methods is that they can be used to analyze the local motion information of the human body, and most methods can overcome changes in the viewing angle. However, this approach requires an accurate two- or three-dimensional human skeleton model, and accurate tracking of key points. However, human modeling and tracking itself remains a challenging problem in the field of computer vision.

### 3.2. Overview of Handcrafted Action Feature for Depth and Skeleton Data

With the application of depth sensors for action recognition, good human detection and pose estimation performance has been achieved using depth data. As a result, most human action recognition methods can be divided into depth sequence-based methods [14,16,24,65,66,67,68], skeleton-based methods [5,18,69,70,71,72,73], and feature fusion methods [17,41,74,75].

The depth sequence-based approach primarily uses motion changes in the depth map of the human body to describe actions. In an RGBD video, the depth data can be seen as a space-time structure composed of depth information. The feature representation of action is the process of feature extraction in this space-time structure, and appearance or motion information regarding depth changes is generally used to describe the action. Yang et al. [14] constructed a supernormal vector feature based on the depth map sequence to represent action. Oreifej et al. [16] extended the HOG feature to the spatiotemporal depth structure, and proposed an orientation histogram feature of the four-dimensional normal vector to represent the appearance information of a three-dimensional spatiotemporal depth structure. Rahmani et al. [67] proposed an action representation method based on the main direction of the depth curved surface. Rotating the perspective of the video in accordance with the main direction, a perspective-independent action feature representation can be calculated, and the principal component direction histogram is used to represent action.

All of the above methods use appearance information to describe human action in depth data. Other methods calculate the motion change from depth information to represent action. Yang et al. [68] proposed a depth motion map (DMM) to project and compress the spatiotemporal depth structure from the front, side, and upper views to form three motion history maps. The HOG feature is then used to represent these motion history maps, and the resulting features are connected in series to describe action. Instead of HOG, Chen et al. [66] used local binary pattern features to describe human action based on DMMs. Chen et al. [76] also analyzed the spatiotemporal depth structure in the front, side, and upper directions. Different from the use of depth information compression, motion trajectory shapes and boundary histogram features of spatiotemporal interest points are extracted and dense sample points and joint points in each view are used to describe the action. In addition, Miao et al. [65] used the discrete cosine variation to compress the depth map and construct action features using transform coefficients.

From depth data, the human skeleton can be estimated quickly and accurately. Shotton et al. [77] proposed a real-time pose estimation method from depth images, which allows rapid human segmentation according to depth. The joint detection problem has been regarded as a simpler per-pixel classification problem. Skeleton-based action recognition methods are another active area of research using depth data. Methods based on the human skeleton sequence use changes in the human joint points between video frames to describe the action, including the position and appearance changes of the joint points. Xia et al. [72] used a 3D joint point histogram to represent the human pose, and modeled the action through a discrete hidden Markov model. Keceli et al. [73] used the Kinect sensor to obtain depth and human skeleton information, and then extracted human action features based on the angle and displacement information of the skeleton joint points. Gowayyed et al. [71] used the histogram of oriented displacements (HOD) to describe the trajectory of joint points, and extracted HOD features from the front, side, and upper views to form a 3D HOD feature. Yang et al. [18] proposed the EigenJoints method, whereby an accumulative motion energy (AME) function is used to select video frames and more informative joint points to model action. Pazhoumanddar et al. [69] used the longest common subsequence algorithm to select high-discriminative power features from the relative motion trajectories of the skeleton to describe the associated action.

Based on the above discussion, methods that use the skeleton joint point trajectory can obtain the corresponding relationship among the joint points from different perspectives. Thus, more robust action features can be extracted in the case of a perspective transformation. However, the performance of these methods depends on the results of human pose estimation. When occlusion occurs in the scene, the joint point estimation will be missing or incorrect, which will affect the action recognition results. Comparing the action features in RGB data with those of depth data, the advantage of RGB data is the abundance of appearance information, although depth data can more accurately describe the motion features of the human body. Research results [5,24,67] show that the methods based on depth information achieve real-time action recognition and better recognition performance than RGB-based methods. Therefore, some researchers have also tried to model human action using multifeature fusion.

Chaaraoui et al. [75] attempted to merge the joint feature and depth information feature to overcome errors in the skeleton feature caused by occlusion and perspective changes. Furthermore, Li et al. [17] proposed a sparse regression learning method based on the joint point group to fuse joint and depth features in modeling actions. Althloothi et al. [78] calculated a spherical harmonics representation of depth information in the frequency domain, and fused this with position information of the skeleton joints through a multikernel learning method to model human actions. In addition to the fusion of depth information and skeletal features, some researchers have attempted to fuse features in RGB data with features in depth data [79]. For example, Liu et al. [74] used genetic algorithms to automatically learn RGB data and depth data fusion features for representing human actions. Jalal et al. [80] merged spatiotemporal features in RGB data and depth data, and Ni et al. [81] proposed a multilevel fusion of RGB and depth data features. Based on object detection, human actions have been modeled by the spatiotemporal features of RGB and depth data. Overall, methods based on data fusion attempt to exploit the advantages between different data to obtain a more robust feature representation. Thus, the following problem is mainly considered during development of most data-fusion-based methods. What is the most effective way of combining data of different types? The common approach involves early and late fusion. The former fusion is carried out at feature level, also known as feature concatenation, which is regarded as the input of the recognition model. In the latter, the fusion is carried at the score level, which fuses the output score of recognition model with different types of data. However, most methods based on multimodal data fusion can provide superior recognition results to those given by single data-based methods. Of course, multimodal data fusion means that processing of larger data volumes is required and the feature dimension is higher. These factors increase the computational complexity of the action recognition algorithm.

### 3.3. Overview of Action Feature Representation Methods Based on Deep Learning

In recent years, the application of deep learning to computer vision has received considerable attention. Many deep learning-based action representation methods have been proposed in the field of human action recognition [19,20,42,45,46,82].

Depending on the structure of the deep learning network, the main representative works can be summarized as discussing methods based on two-stream convolutional networks [19], those based on 3D convolutional networks [20], and those based on long short-term memory (LSTM) [83].

In a two-stream convolutional network, the optical flow information is calculated from the image sequence. The image and optical flow sequence are respectively used as the input to the two convolutional neural networks (CNNs) during the model training process. Fusion occurs in the last classification layer of the network. The inputs of the two-stream network are a single-frame image and a multioptical flow frame image stack, and the network applies 2D image convolution. In contrast, a 3D convolution network regards the video as a 3D space-time structure, and uses a 3D convolution method to learn human action features.

Many studies attempting to improve the performance of these two network structures have been reported. Some representative extensions of the two-stream convolution network include the work of Zhang et al. [82], who used the motion vector in the video stream instead of the optical flow sequence to improve the calculation speed and realize real-time human action recognition. Feichtenhofer et al. [84] changed the process of spatial and temporal information fusion from the original final classification layer to the middle of the network, further improving the action recognition accuracy. The input, convolutional network structure, and training strategy of the two-stream convolutional network have been discussed in detail by Wang et al. [85], and a temporal segment network (TSN) was proposed to further improve the results of the two-stream convolution network. In addition, Lan [86] and Zhou [87] also enhanced the recognition results of TSN. Similarly, based on the idea of 3D (Three Dimension) convolutional networks, many researchers have attempted to extend different 2D convolutional networks to 3D spatiotemporal structures for the learning and recognition of human action features. Carreira et al. [88] extended the network structure of inception-V1 from two to three dimensions and proposed the two-stream inflated 3D ConvNet for action recognition. Diba et al [89] extended DenseNet and proposed the temporal 3D ConvNet, while Zhu et al. [90] extended the pooling operation from the original spatial dimension to three space-time dimensions, and expanded the two-stream convolution network to a three-dimensional structure.

Another important human action recognition method involves use of LSTM with CNNs [83,88,91,92]. In Carreira’ work [88], five different architectures, including an LSTM with CNN, 3D ConvNet, a two-stream network, a two-stream inflated 3D ConvNet, and a 3D-Fused two-stream network, are compared. Different from two-stream and 3D convolutional networks, in which action is modeled by various convolutional temporal feature pooling architectures, LSTM-based methods regard a video as an ordered sequence of frames. Human action can then be represented by the feature changes of each frame. Ng et al. [91] proposed a recurrent neural network to recognize human action that connects the LSTM cell with the output of the underlying CNN. Further, Qiu et al. [92] proposed a new architecture named Pseudo-3D Residual Net (P3D ResNet), which composes each block in a different ResNet placement. Donahue et al. [83] proposed long-term recurrent convolutional networks to map variable-length video frames to variable length outputs (e.g., action descriptive text) rather than a simple action category. Liu et al. [42] proposed a spatiotemporal LSTM model for 3D human action recognition, which extends recurrent neural networks (RNNs) to spatiotemporal domains to analyze the hidden sources of action-related information. Song et al. [93] constructed the action recognition model on top of the RNN with LSTM, which used different levels of attention to learn the discriminative joints of the skeleton within each input frame.

From the modality of the input data, deep learning methods can learn human action features from a variety of modal data. The above methods are based on image data or optical flow data, and model human actions from the appearance information and motion information of temporal and spatial changes. In addition, Wang et al. [94] used a convolutional network to learn action features based on depth image sequences. Ye et al. [95] used 3D convolutional networks to embed time series information into dense motion trajectories to model action. Yan et al. [46] used graph-oriented CNN to model the underlying relationships between skeleton joints with a graph. Zhu et al. [96] attempted to integrate the calculation of the optical flow sequence into the network, and merge the original image and optical flow sequence to represent human action. Although the action features learned by deep learning methods offer superior recognition performance to handcrafted action features, some problems also exist as regards recognition of human actions based on deep learning, especially multimodal data fusion in deep learning methods. Most of the above deep learning-based methods focus on the problem of action feature learning from different modality data; however, few works consider multimodal data fusion. Effective combination of multimodal data, such as RGB, optical flow, depth, and skeleton data, remains an open issue in human action recognition and deep learning. This topic is also a prominent research direction in the field of human action recognition.

### 3.4. Performance Evaluation Criteria and Datset

There are many datasets for evaluation of algorithm performance in the context of human action recognition. The confusion matrix and recognition accuracy are the commonly used evaluation criteria. The confusion matrix indicates the detailed results concerning recognition between each category. The recognition accuracy is the ratio of the number of correctly recognized data elements to the total number of test data elements. Details of popular datasets that contain more than 300 samples are presented in Table 1. The recognition accuracies of the popular methods for these datasets are presented in Table 2 and Table 3.

Table 2 and Table 3 show the methods exhibiting good recognition performance for each year on these datasets. From these tables, it is apparent that the deep learning-based methods have better recognition performance than those based on handcrafted features developed in the last three years, especially for the action recognition problem involving RGB data. Following application of deep learning methods for action feature learning, the action recognition accuracy improved significantly, such that the recognition accuracy for the UCF 101 dataset is improved from 89.1% to 97.9%, and that for the HMDB51 dataset is improved from 65.1% to 80.2%.

Among these RGB datasets, HMDB51 and UCF 101 are the most commonly used to evaluate the proposed approaches. Studies on almost all the most recent deep learning-based methods have employed these two datasets to verify algorithm effectiveness. The recognition accuracy for the UCF 101 dataset has exceeded 97%; however, equivalent accuracy has not yet been achieved on HMDB51. Note that the Kinetics dataset is a new and more challenging dataset for human action recognition. Deep learning-based methods have been less frequently applied to its RGBD and skeleton datasets than its RGB dataset. One of the main reasons for this is that these datasets are small-scale. However, with the development of large-scale and challenging RGBD and skeleton datasets, for example, the NTU RGB+D dataset, deep-leaning-based methods for depth and skeleton data are becoming the primary research topic in the field of human action recognition.

## 4. Overview of Interaction Recognition Methods

In complex scenes, such as those acquired by intelligent video surveillance, abnormal actions are often related to the attachments carried by humans. This is also the problem of interaction recognition in human action recognition research, which is a challenging task. Studies on interaction recognition have become a popular research topic in recent years. Because objects and poses provide rich action features for interaction recognition, many methods are based on image data [25]. In early studies [112,113,114], researchers attempted to use the relationship between the object, human pose, and action to integrate object detection, pose estimation, and action analysis methods into one framework. The middle-level semantic feature of interaction is extracted based on the object detection and pose estimation results. A summary of these methods reveals that the design of the interaction feature mainly follows a set of principles:

(1) Local interaction features should be dense enough to represent information at various locations in the image.
(2) The model of interaction between the human and the object(s) is based on the structure of body parts.
(3) The core of the interaction model is the co-occurrence and position relationship between the human body and the object(s).
(4) Features with higher discriminative power are selected from dense features.

Other researchers have attempted to use depth information and skeleton data to model human–object interactions [26,115,116]. Meng et al. [115] expressed the interaction based on the joint points detected in the depth information, the relative position changes between these joint points, and the relative positional changes between the joint points and the objects. Koppula et al. [116] used a Markov random field to model the interaction, where the graph nodes represent the object and the human motion and the edges represent the relationship.

With the development of deep learning technology, interaction recognition methods based on deep learning have attracted attention. However, a large number of datasets are required for the application of deep learning. Le et al. [28] provided the first large interaction recognition dataset named TUHIO. Chao et al. [27] released the HOIC dataset in 2015, including more than 600 interactions across 47,774 images. The synonymous interactions of TUHIO have been integrated into HOIC. Gupta et al. [117] provided the V-COCO dataset, which is a subset of the COCO dataset including 10,346 images and 16,199 human instances. In that dataset, each person is annotated with a binary label vector for 26 different actions. The above three datasets are image-based. Yu et al. [118] proposed a video-based dataset for online human object interaction recognition named ORGBD, which contains three sets of depth sequences collected using a Kinect device. The first set contains 16 subjects and each subject performs every action twice. The second set contains eight new subjects recorded in different environments from the first. Each sequence in the third set consists of multiple unsegmented actions. Furthermore, Meng et al. [26] constructed a new video-based human object interaction recognition dataset in 2018, called Lille Douai.

Based on these data, Chao et al. [119] proposed the human body and object region-based CNN to analyze the relationship between humans and objects, with a deep neural network used to detect the interaction. Mallya et al. [120] calculated human and object features from pretrained CNNs, and merged them with pretrained human and object relationship models learned from the HIOC dataset to detect interactions. Gkioxari et al. [121] hypothesized that the appearance of the human body, such as posture, clothing, and action, is closely related to the position of the object. They proposed an interactive network (InterNet) to model the relationship between the human body and objects for specific actions. For interaction recognition, the strategy of deep learning methods is to automatically learn the relationship model between the human body and objects based on human detection and the available data. With limited data, especially in the intelligent monitoring environment, abnormal actors will deliberately obscure the objects being carried, resulting in errors in the position relationship between the human body and the objects caused by the camera imaging. These issues mean that interaction recognition remains a challenging problem.

## 5. Overview of Human Action Detection Methods

Compared to action classification, action detection is a more challenging task in the context of human action recognition. In early research, action detection mainly used object tracking and sliding windows to locate the action position [50]. However, because of the difficulty of object tracking and the complexity of sliding windows, action detection is still a difficult task in human action recognition. Building on the strong performance of dense trajectories (DT) in action classification, researchers have used DT features to locate actions in the time dimension [51,111,122]. Van et al. [3] also used DT features to extend the action location from the time dimension to the space-time dimension, resulting in faster processing speeds. At the same time, depth sensors have a natural advantage in human location and skeleton extraction, so action detection based on depth sensors is simpler and more effective than that using RGB data [31].

With the successful application of deep learning methods in multiobject detection, some methods for detecting action in video based on deep learning have been proposed. The basic idea of these methods is to extend the network of object classification to action detection problems, including R-CNN networks, 3D convolutional networks, and recurrent neural networks. Peng et al. [29] proposed multiregion two-stream R-CNN networks to locate action in the time dimension, while Shou et al. [123] used three 3D convolutional networks to detect action. First, the candidate region extraction network is used to segment video in the time dimension. Based on the extracted candidate segmentation regions, the classification network and the location network are then used to detect action. Zhao et al. [47] regarded action detection as the problem of segmenting the video content, and proposed structured segment networks to implement the segmentation of action in the time dimension. Liu et al. [30] proposed a multimodality multitask recurrent neural network to model action in RGB and skeleton data, and integrated action classification and localization into a unified network to recognize action. Zhu et al. [4] fused the context among video frames into a long-term recurrent convolutional network, and proposed spatial-temporal convolution regression networks for action detection. In addition, Yu et al. [124] calculated the similarity scores of each region and action using appearance and motion features. Based on this, the problem of spatiotemporal candidate region extraction can be regarded as the extraction of the largest set. Weinzaepfel et al. [125] calculated the relevance scores between each frame and action based on CNN features of appearance and motion, and tracked the spatiotemporal motion histograms to locate human action.

In action detection research, the ActivityNet challenge is very famous task. This challenge was first hosted during the 2016 Conference on Computer Vision and Pattern Recognition and focused on the recognition of daily life, high-level, goal-oriented activities. There are three tasks based on the ActivityNet dataset: action temporal proposal, action localization, and action caption. The ActivityNet dataset [126] is a large-scale video benchmark for human activity understanding, which contains 200 action classes and 100 untrimmed videos per class, yielding a total of 648 hours. The results obtained through application of different methods to these three tasks are provided on the ActivityNet challenge webpage [127].

Although the research on action detection has already achieved some promising results, most of the work has focused on locating the position of the action in the time dimension, and most approaches have a high computational complexity. Action detection in real applications involves the semantic segmentation of the video content. This technology is still far from being mature.

## 6. Conclusions and Discussion

In this work we have provided a comprehensive overview of human action recognition research and discussed future research directions for those seeking to enter the field. Although there have been many excellent studies on human action recognition, confounding factors, such as the diversity and complexity of body postures, occlusion, and background clutter, mean that human action recognition in real scenes remains challenging in the context of understanding video streams. In this paper, we have reviewed human action recognition methods and provided a comprehensive overview of recent approaches to human action recognition research, including hand-designed action features in RGB and depth data, deep learning-based action feature representation methods, human–object interaction recognition techniques, and action detection methods. After summarizing the literature relevant to each research direction in the field of action recognition, the main effective methods were introduced, to rapidly familiarize researchers with the relevant research areas. The primary conclusions of this study on human action recognition research are provided below.

(1) In action recognition research, suitable data to capture the action must first be selected. In addition, a reasonable algorithm should be used to recognize human action.
(2) For action feature learning problems, deep learning-based methods have superior performance.
(3) In addition to action classification of primitive and single-person actions, interaction recognition, and action detection have become the new prominent research topics.

The performance of deep learning in human action representation, interaction recognition, and action detection has been validated in certain circumstances. However, in complex scenes such as intelligent video surveillance, there are many issues with feature learning under multimodal data, interaction recognition, and spatial and temporal action localization. Given the current state of research, human action recognition based on deep learning in intelligent video surveillance should address the following problems.

(1) Multimodal visual perception and action representation problems based on multimodal fusion methods. In current research, based on accurate depth information and skeleton data, human action features can be efficiently studied. However, in most real scenes, the data acquisition platform can only provide RGB data. While depth sensors can be applied outdoors, problems with accuracy and cost mean they are not suitable for monitoring scenarios. Obtaining accurate modal data, such as accurate depth information and skeleton data from existing RGB data, is not only the basis of efficient action recognition, but also has important applications in many visual analysis tasks. Based on RGB data, depth data, and skeleton data, the integration of multimodal data is a key issue in behavior recognition research.
(2) Interaction recognition problems. The recognition of interactions between humans and objects has a high level of semantic information, such as carrying dangerous items, legacy items, and waving equipment. Modeling the interaction between humans and articles based on multimodal data, and quickly analyzing the interaction, is not currently possible to an appropriate level of accuracy. This will be an important direction of future research on human action recognition.
(3) Fast action detection in the spatiotemporal dimension. Research on human action recognition is more about classifying segmented video content. Although there have been some attempts to discuss how the action is located in the time and space dimensions, the effect and speed are below current application requirements. Analyzing the features of different information based on multimodal data perception, and achieving fast and accurate action detection, are the key issues for the successful application of human action recognition. In recent years, several methods have been proposed, but this is another challenge that is yet unresolved.

## Figures and Tables

**Table 1:** The popular dataset of human action recognition.

Dataset Name	Color	Depth	Skeleton	Samples	Classes
Hollywood2 [97]	√	×	×	1707	12
HMDB51 [98]	√	×	×	6766	51
Olympic Sports [99]	√	×	×	783	16
UCF50 [100]	√	×	×	6618	50
UCF101 [101]	√	×	×	13,320	101
Kinetics [102]	√	×	×	306,245	400
MSR-Action3D [103]	×	√	√	567	20
MSR-Daily Activity [104]	√	√	√	320	16
Northwestern-UCLA [105]	√	√	√	1475	10
UTD-MHAD [106]	√	√	√	861	27
RGBD-HuDaAct [107]	√	√	×	1189	13
NTU RGB+D [108]	√	√	√	56,880	60

**Table 2:** Recognition accuracies of methods on RGB datasets. The superscript D indicates a deep learning-based method.

Methods	Year	Hollywood2	HMDB51	Olympic Sports	UCF50	UCF101	Kinetic
[82]^D^	2018					86.4%	
[46]^D^	2018						81.5%
[61]^D^	2018	68.1%		94%			
[96]^D^	2017		78.7%			97.1%	
[89]^D^	2017		63.5%			93.2%	
[86]^D^	2017		75%			95.3%	
[102]^D^	2017						79%
[90]^D^	2017		74.8%			95.8%	
[88]^D^	2017		80.2%			97.9%	
[95]^D^	2016		55.2%			85.4%	
[85]^D^	2016		69.4%			94.2%	
[63]	2016	66.8%	60.1%	90.4%	91.7%	86%	
[29]^D^	2016			94.8%		78.86%	
[84]^D^	2016		65.4%			92.5%	
[45]^D^	2015		63.2%			91.5%	
[109]	2016		61.7%			88.3%	
[110]	2015	68%	65.1%	91.4%	94.4%	89.1%	
[20]^D^	2015		49.9%			85.2%	79.5%
[40]	2015	70%	61.8%				
[57]	2014		37.3%	86.04%	70.1%		
[50]	2014		57.2%			85.9%	
[19]	2014		59.4%			88%	81.3%
[62]	2014	54.4%	41.3%	85.5%			
[100]	2013		27.02%		68.20%		
[111]	2013	49.3%					

**Table 3:** Recognition accuracies of methods on RGBD (Red, Green, Blue Depth) and skeleton datasets. The superscript D indicates a deep learning-based method.

Methods		MSR-Action3D	MSR-Daily Activity	Northwestern-UCLA	UTD-MHAD	NTU RGB+D
[31]	2018	96.2%				
[93]^D^	2018					73.4%
[46]^D^	2018					30.7%
[80]	2017	93.3	94.1%			
[108]^D^	2016					62.93%
[42]^D^	2016				100%	69.2%
[94]^D^	2015	100%	81.88%			
[69]	2015	91.2%				
[76]	2015	94.9%	83.8%			
[106]	2015				79.1%	
[14]	2014	93.09%	86.25%			31.82%
[105]	2014		73.1%	81.6%		
[18]	2014	82.30%				
[70]	2014	92.46%				50.1%
[67]	2014	88.82%	81.25%			
[78]	2014	94.4%	93.1%			
[16]	2013	88.89%				30.56%
[74]	2013		85.6%			
[71]	2013	91.26%				
[75]	2013	91.80%				

## References

[1] Aggarwal J.K., Ryoo M.S. (2011). Human activity analysis: A review. ACM Comput. Surv..

[2] Ziaeefard M., Bergevin R. (2015). Semantic human activity recognition: A literature review. Pattern Recognit..

[3] Van Gemert J.C., Jain M., Gati E., Snoek C.G. APT: Action localization proposals from dense trajectories. Proceedings of the British Machine Vision Conference 2015: BMVC 2015.

[4] Zhu H., Vial R., Lu S. Tornado: A spatio-temporal convolutional regression network for video action proposal. Proceedings of the CVPR.

[5] Papadopoulos G.T., Axenopoulos A., Daras P. Real-time skeleton-tracking-based human action recognition using kinect data. Proceedings of the International Conference on Multimedia Modeling.

[6] Presti L.L., Cascia M.L. (2016). 3D Skeleton-based Human Action Classification: A Survey. Pattern Recognit..

[7] Paul S.N., Singh Y.J. (2014). Survey on Video Analysis of Human Walking Motion. Int. J. Signal Process. Image Process. Pattern Recognit..

[8] Bengio Y., Courville A., Vincent P. (2013). Representation Learning: A Review and New Perspectives. IEEE Trans. Pattern Anal. Mach. Intell..

[9] Dawn D.D., Shaikh S.H. (2016). A comprehensive survey of human action recognition with spatio-temporal interest point (STIP) detector. Vis. Comput..

[10] Nguyen T.V., Song Z., Yan S.C. (2015). STAP: Spatial-Temporal Attention-Aware Pooling for Action Recognition. IEEE Trans. Circ. Syst. Video Technol..

[11] Shao L., Zhen X.T., Tao D.C., Li X.L. (2014). Spatio-Temporal Laplacian Pyramid Coding for Action Recognition. IEEE Trans. Cybern..

[12] Burghouts G.J., Schutte K., ten Hove R.J.M., van den Broek S.P., Baan J., Rajadell O., van Huis J.R., van Rest J., Hanckmann P., Bouma H. (2014). Instantaneous threat detection based on a semantic representation of activities, zones and trajectories. Signal Image Video Process..

[13] Wang H., Schmid C. Action recognition with improved trajectories. Proceedings of the ICCV.

[14] Yang X., Tian Y.L. Super Normal Vector for Activity Recognition Using Depth Sequences. Proceedings of the 2014 IEEE Conference on Computer Vision and Pattern Recognition.

[15] Ye M., Zhang Q., Wang L., Zhu J., Yang R., Gall J. A survey on human motion analysis from depth data. Proceedings of the Dagstuhl 2012 Seminar on Time-of-Flight Imaging: Sensors, Algorithms, and Applications and Workshop on Imaging New Modalities, GCPR 2013.

[16] Oreifej O., Liu Z. HON4D: Histogram of Oriented 4D Normals for Activity Recognition from Depth Sequences. Proceedings of the Computer Vision and Pattern Recognition.

[17] Li M., Leung H., Shum H.P.H. Human action recognition via skeletal and depth based feature fusion. Proceedings of the Motion in Games 2016.

[18] Yang X., Tian Y.L. (2014). Effective 3D action recognition using EigenJoints. J. Vis. Commun. Image Represent..

[19] Simonyan K., Zisserman A. Two-Stream Convolutional Networks for Action Recognition in Videos. Proceedings of the NIPS 2014: Neural Information Processing Systems Conference.

[20] Tran D., Bourdev L.D., Fergus R., Torresani L., Paluri M. Learning Spatiotemporal Features with 3D Convolutional Networks. Proceedings of the IEEE International Conference on Computer Vision 2015.

[21] Güler R.A., Neverova N., Kokkinos I. (2018). DensePose: Dense Human Pose Estimation in the Wild. arXiv.

[22] Fang H.-S., Xie S., Tai Y.-W., Lu C. RMPE: Regional Multi-Person Pose Estimation. Proceedings of the 2017 IEEE International Conference on Computer Vision Workshops (ICCVW).

[23] Cao Z., Simon T., Wei S.-E., Sheikh Y. Realtime multi-person 2d pose estimation using part affinity fields. Proceedings of the IEEE Conference on Computer Vision and Pattern Recognition 2017.

[24] Chen C., Liu K., Kehtarnavaz N. (2016). Real-time human action recognition based on depth motion maps. J. Real-Time Image Process..

[25] Guo G., Lai A. (2014). A survey on still image based human action recognition. Pattern Recognit..

[26] Meng M., Drira H., Boonaert J. (2018). Distances evolution analysis for online and off-line human object interaction recognition. Image Vis. Comput..

[27] Chao Y., Wang Z., He Y., Wang J., Deng J. HICO: A Benchmark for Recognizing Human-Object Interactions in Images. Proceedings of the International Conference on Computer Vision.

[28] Le D.-T., Uijlings J., Bernardi R. Tuhoi: Trento universal human object interaction dataset. Proceedings of the Third Workshop on Vision and Language.

[29] Peng X., Schmid C. Multi-region two-stream R-CNN for action detection. Proceedings of the Computer Vision—ECCV 2016: 14th European Conference.

[30] Liu J., Li Y., Song S., Xing J., Lan C., Zeng W. (2018). Multi-Modality Multi-Task Recurrent Neural Network for Online Action Detection. IEEE Trans. Circ. Syst. Video Technol..

[31] Patrona F., Chatzitofis A., Zarpalas D., Daras P. (2018). Motion analysis: Action detection, recognition and evaluation based on motion capture data. Pattern Recognit..

[32] Herath S., Harandi M., Porikli F. (2017). Going deeper into action recognition: A survey. Image Vis. Comput..

[33] Subetha T., Chitrakala S. A survey on human activity recognition from videos. Proceedings of the IEEE 2016 International Conference on Information Communication and Embedded Systems.

[34] Vishwakarma S., Agrawal A. (2013). A survey on activity recognition and behavior understanding in video surveillance. Vis. Comput..

[35] Wang J., Chen Y., Hao S., Peng X., Hu L. (2018). Deep learning for sensor-based activity recognition: A Survey. Pattern Recognit. Lett..

[36] Yu K., Yun F. (2018). Human Action Recognition and Prediction: A Survey. arXiv.

[37] Liu A., Xu N., Nie W., Su Y., Wong Y., Kankanhalli M.S. (2017). Benchmarking a Multimodal and Multiview and Interactive Dataset for Human Action Recognition. IEEE Trans. Syst. Man Cybern..

[38] Liu A., Su Y., Nie W., Kankanhalli M.S. (2017). Hierarchical Clustering Multi-Task Learning for Joint Human Action Grouping and Recognition. IEEE Trans. Pattern Anal..

[39] Gao Z., Zhang Y., Zhang H., Xue Y., Xu G. (2016). Multi-dimensional human action recognition model based on image set and group sparisty. Neurocomputing.

[40] Fernando B., Gavves E., Oramas M.J., Ghodrati A., Tuytelaars T. Modeling video evolution for action recognition. Proceedings of the Computer Vision and Pattern Recognition.

[41] Zhang J., Li W., Ogunbona P., Wang P., Tang C. (2016). RGB-D-based action recognition datasets. Pattern Recognit..

[42] Liu J., Shahroudy A., Xu D., Wang G. Spatio-Temporal LSTM with Trust Gates for 3D Human Action Recognition. Proceedings of the ECCV.

[43] Zhang H.-B., Lei Q., Zhong B.-N., Du J.-X., Peng J., Hsiao T.-C., Chen D.-S. (2016). Multi-Surface Analysis for Human Action Recognition in Video.

[44] Zhang Z., Liu S., Han L., Shao Y., Zhou W., Mu J. (2015). Human action recognition using salient region detection in complex scenes. Proceedings of Third International Conference on Communications, Signal Processing, and Systems.

[45] Wang L., Qiao Y., Tang X. Action recognition with trajectory-pooled deep-convolutional descriptors. Proceedings of the Computer Vision and Pattern Recognition.

[46] Yan S., Xiong Y., Lin D. (2018). Spatial Temporal Graph Convolutional Networks for Skeleton-Based Action Recognition. arXiv.

[47] Zhao Y., Xiong Y., Wang L., Wu Z., Tang X., Lin D. Temporal action detection with structured segment networks. Proceedings of the IEEE International Conference on Computer Vision (ICCV).

[48] Zhou Z., Shi F., Wu W. (2015). Learning Spatial and Temporal Extents of Human Actions for Action Detection. IEEE Trans. Multimed..

[49] Zhang H.B., Li S.Z., Chen S.Y., Su S.Z., Lin X.M., Cao D.L. (2015). Locating and recognizing multiple human actions by searching for maximum score subsequences. Signal Image Video Process..

[50] Shu Z., Yun K., Samaras D. (2014). Action Detection with Improved Dense Trajectories and Sliding Window. Proceedings of ECCV.

[51] Oneata D., Verbeek J.J., Schmid C. Efficient Action Localization with Approximately Normalized Fisher Vectors. Proceedings of the 2014 IEEE Conference on Computer Vision and Pattern Recognition.

[52] Chakraborty B.K., Sarma D., Bhuyan M.K., MacDorman K.F. (2018). Review of constraints on vision-based gesture recognition for human–computer interaction. IET Comput. Vis..

[53] Ibrahim M.S., Muralidharan S., Deng Z., Vahdat A., Mori G. A Hierarchical Deep Temporal Model for Group Activity Recognition. Proceedings of the 2016 IEEE Conference on Computer Vision and Pattern Recognition (CVPR).

[54] Bobick A.F., Davis J.W. (2001). The recognition of human movement using temporal templates. IEEE Trans. Pattern Anal..

[55] Zhang Z., Hu Y., Chan S., Chia L. (2008). Motion Context: A New Representation for Human Action Recognition. Proceedings of European Conference on Computer Vision.

[56] Klaser A., Marszalek M., Schmid C. A Spatio-Temporal Descriptor Based on 3D-Gradients. Proceedings of the British Machine Vision Conference.

[57] Somasundaram G., Cherian A., Morellas V., Papanikolopoulos N. (2014). Action recognition using global spatio-temporal features derived from sparse representations. Comput. Vis. Image Underst..

[58] Laptev I. (2005). On space-time interest points. Int. J. Comput. Vis..

[59] Chakraborty B., Holte M.B., Moeslund T.B., Gonzalez J. (2012). Selective spatio-temporal interest points. Comput. Vis. Image Underst..

[60] Peng X., Wang L., Wang X., Qiao Y. (2016). Bag of visual words and fusion methods for action recognition: Comprehensive study and good practice. Comput. Vis. Image Underst..

[61] Nazir S., Yousaf M.H., Velastin S.A. (2018). Evaluating a bag-of-visual features approach using spatio-temporal features for action recognition. Comput. Electr. Eng..

[62] Gaidon A., Harchaoui Z., Schmid C. (2014). Activity representation with motion hierarchies. Int. J. Comput. Vis..

[63] Wang H., Oneata D., Verbeek J.J., Schmid C. (2016). A Robust and Efficient Video Representation for Action Recognition. Int. J. Comput. Vis..

[64] Peng X., Zou C., Qiao Y., Peng Q. (2014). Action Recognition with Stacked Fisher Vectors. Proceedings of European Conference on Computer Vision.

[65] Miao J., Jia X., Mathew R., Xu X., Taubman D., Qing C. Efficient action recognition from compressed depth maps. Proceedings of the International Conference on Image Processing.

[66] Chen C., Jafari R., Kehtarnavaz N. Action Recognition from Depth Sequences Using Depth Motion Maps-Based Local Binary Patterns. Proceedings of the Workshop on Applications of Computer Vision.

[67] Rahmani H., Mahmood A., Huynh D.Q., Mian A. Real time action recognition using histograms of depth gradients and random decision forests. Proceedings of the IEEE Winter Conference on Applications of Computer Vision.

[68] Yang X., Zhang C., Tian Y.L. Recognizing actions using depth motion maps-based histograms of oriented gradients. Proceedings of the ACM International Conference on Multimedia.

[69] Pazhoumanddar H., Lam C.P., Masek M. (2015). Joint movement similarities for robust 3D action recognition using skeletal data. J. Vis. Commun. Image Represent..

[70] Vemulapalli R., Arrate F., Chellappa R. Human Action Recognition by Representing 3D Skeletons as Points in a Lie Group. Proceedings of the Computer Vision and Pattern Recognition.

[71] Gowayyed M.A., Torki M., Hussein M.E., Elsaban M. Histogram of oriented displacements (HOD): Describing trajectories of human joints for action recognition. Proceedings of the International Joint Conference on Artificial Intelligence.

[72] Xia L., Chen C., Aggarwal J.K. View invariant human action recognition using histograms of 3D joints. Proceedings of the Computer Vision and Pattern Recognition.

[73] Keceli A.S., Can A.B. (2014). Recognition of Basic Human Actions using Depth Information. Int. J. Pattern Recognit. Artif. Intell..

[74] Liu L., Shao L. Learning discriminative representations from RGB-D video data. Proceedings of the International Joint Conference on Artificial Intelligence.

[75] Chaaraoui A.A., Padillalopez J.R., Florezrevuelta F. Fusion of Skeletal and Silhouette-Based Features for Human Action Recognition with RGB-D Devices. Proceedings of the 2013 IEEE International Conference on Computer Vision Workshops.

[76] Chen W., Guo G. (2015). TriViews: A general framework to use 3D depth data effectively for action recognition. J. Vis. Commun. Image Represent..

[77] Shotton J., Sharp T., Fitzgibbon A., Blake A., Cook M., Kipman A., Finocchio M., Moore R. (2013). Real-Time human pose recognition in parts from single depth images. Commun. ACM.

[78] Althloothi S., Mahoor M.H., Zhang X., Voyles R.M. (2014). Human activity recognition using multi-features and multiple kernel learning. Pattern Recognit..

[79] Sanchezriera J., Hua K., Hsiao Y., Lim T., Hidayati S.C., Cheng W. (2016). A comparative study of data fusion for RGB-D based visual recognition. Pattern Recognit. Lett..

[80] Jalal A., Kim Y., Kim Y., Kamal S., Kim D. (2017). Robust human activity recognition from depth video using spatiotemporal multi-fused features. Pattern Recognit..

[81] Ni B., Pei Y., Moulin P., Yan S. (2013). Multilevel Depth and Image Fusion for Human Activity Detection. IEEE Trans. Syst. Man Cybern..

[82] Zhang B., Wang L., Wang Z., Qiao Y., Wang H. (2018). Real-Time Action Recognition With Deeply Transferred Motion Vector CNNs. IEEE Trans. Image Process..

[83] Donahue J., Hendricks L.A., Rohrbach M., Venugopalan S., Guadarrama S., Saenko K., Darrell T. (2017). Long-Term Recurrent Convolutional Networks for Visual Recognition and Description. IEEE Trans. Pattern Anal..

[84] Feichtenhofer C., Pinz A., Zisserman A. Convolutional Two-Stream Network Fusion for Video Action Recognition. Proceedings of the 2016 IEEE Conference on Computer Vision and Pattern Recognition (CVPR).

[85] Wang L., Xiong Y., Wang Z., Qiao Y., Lin D., Tang X., Gool L.V. (2016). Temporal Segment Networks: Towards Good Practices for Deep Action Recognition. Proceedings of ECCV.

[86] Lan Z., Zhu Y., Hauptmann A.G., Newsam S. Deep Local Video Feature for Action Recognition. Proceedings of the 2017 IEEE Conference on Computer Vision and Pattern Recognition Workshops (CVPRW).

[87] Zhou B., Andonian A., Torralba A. (2017). Temporal Relational Reasoning in Videos. arXiv.

[88] Carreira J., Zisserman A. Quo vadis, action recognition? A new model and the kinetics dataset. Proceedings of the 2017 Conference on Computer Vision and Pattern Recognition.

[89] Diba A., Fayyaz M., Sharma V., Karami A.H., Arzani M.M., Yousefzadeh R., Van Gool L. (2017). Temporal 3D ConvNets: New Architecture and Transfer Learning for Video Classification. arXiv.

[90] Zhu J., Zou W., Zhu Z. (2017). End-to-end Video-Level Representation Learning for Action Recognition. arXiv.

[91] Ng Y.H., Hausknecht M., Vijayanarasimhan S., Vinyals O., Monga R., Toderici G. Beyond Short Snippets: Deep Networks for Video Classification. Proceedings of the Computer Vision & Pattern Recognition.

[92] Qiu Z., Yao T., Mei T. Learning spatio-temporal representation with pseudo-3d residual networks. Proceedings of the 2017 IEEE International Conference on Computer Vision (ICCV).

[93] Song S., Lan C., Xing J., Zeng W., Liu J. (2018). Spatio-Temporal Attention Based LSTM Networks for 3D Action Recognition and Detection. IEEE Trans. Image Process..

[94] Wang P., Li W., Gao Z., Zhang J., Tang C., Ogunbona P. (2015). Deep Convolutional Neural Networks for Action Recognition Using Depth Map Sequences. arXiv.

[95] Ye Y., Tian Y. Embedding Sequential Information into Spatiotemporal Features for Action Recognition. Proceedings of the Computer Vision and Pattern Recognition.

[96] Zhu Y., Lan Z., Newsam S., Hauptmann A.G. (2017). Hidden two-stream convolutional networks for action recognition. arXiv.

[97] Marszalek M., Laptev I., Schmid C. Actions in context. Proceedings of the 2009 IEEE Computer Society Conference on Computer Vision and Pattern Recognition Workshops, CVPR Workshops 2009.

[98] Kuehne H., Jhuang H., Stiefelhagen R., Serre T. (2013). HMDB51: A Large Video Database for Human Motion Recognition.

[99] Niebles J.C., Chen C.W., Li F.F. (2010). Modeling Temporal Structure of Decomposable Motion Segments for Activity Classification. Proceedings of European Conference on Computer Vision.

[100] Reddy K.K., Shah M. (2013). Recognizing 50 human action categories of web videos. Mach. Vis. Appl..

[101] Soomro K., Zamir A.R., Shah M. (2012). UCF101: A Dataset of 101 Human Actions Classes from Videos in The Wild. arXiv.

[102] Kay W., Carreira J., Simonyan K., Zhang B., Hillier C., Vijayanarasimhan S., Viola F., Green T., Back T., Natsev P. (2017). The Kinetics Human Action Video Dataset. arXiv.

[103] Li W., Zhang Z., Liu Z. Action recognition based on a bag of 3D points. Proceedings of the 2010 IEEE Computer Society Conference on Computer Vision and Pattern Recognition—Workshops, CVPRW 2010.

[104] Wu Y. Mining actionlet ensemble for action recognition with depth cameras. Proceedings of the 2012 IEEE Conference on Computer Vision and Pattern Recognition (CVPR).

[105] Wang J., Nie X., Xia Y., Wu Y., Zhu S.C. Cross-view Action Modeling, Learning and Recognition. Proceedings of the Computer Vision and Pattern Recognition.

[106] Chen C., Jafari R., Kehtarnavaz N. UTD-MHAD: A multimodal dataset for human action recognition utilizing a depth camera and a wearable inertial sensor. Proceedings of the 2015 IEEE International Conference on Image Processing (ICIP).

[107] Ni B., Wang G., Moulin P. RGBD-HuDaAct: A color-depth video database for human daily activity recognition. Proceedings of the ICCV Workshops.

[108] Shahroudy A., Liu J., Ng T.-T., Wang G. NTU RGB+D: A Large Scale Dataset for 3D Human Activity Analysis. Proceedings of the IEEE Conference on Computer Vision and Pattern Recognition 2016.

[109] Wang L.M., Qiao Y., Tang X.O. (2016). MoFAP: A Multi-level Representation for Action Recognition. Int. J. Comput. Vis..

[110] Lan Z., Ming L., Li X., Hauptmann A.G., Raj B. Beyond Gaussian Pyramid: Multi-skip Feature Stacking for Action Recognition. Proceedings of the IEEE Conference on Computer Vision and Pattern Recognition 2015.

[111] Gaidon A., Harchaoui Z., Schmid C. (2013). Temporal localization of actions with actoms. IEEE Trans. Pattern Anal..

[112] Prest A., Ferrari V., Schmid C. (2013). Explicit Modeling of Human-Object Interactions in Realistic Videos. IEEE Trans. Pattern Anal..

[113] Yao B., Fei-Fei L. (2012). Recognizing human-object interactions in still images by modeling the mutual context of objects and human poses. IEEE Trans. Pattern Anal..

[114] Desai C., Ramanan D. (2012). Detecting actions, poses, and objects with relational phraselets. Proceedings of the European Conference on Computer Vision.

[115] Meng M., Drira H., Daoudi M., Boonaert J. Human Object Interaction Recognition Using Rate-Invariant Shape Analysis of Inter Joint Distances Trajectories. Proceedings of the Computer Vision and Pattern Recognition Workshops.

[116] Koppula H.S., Gupta R., Saxena A. (2013). Learning human activities and object affordances from rgb-d videos. Int. J. Robot. Res..

[117] Gupta S., Malik J. (2015). Visual Semantic Role Labeling. arXiv.

[118] Yu G., Liu Z., Yuan J. (2014). Discriminative Orderlet Mining for Real-Time Recognition of Human-Object Interaction.

[119] Chao Y.-W., Liu Y., Liu X., Zeng H., Deng J. (2017). Learning to Detect Human-Object Interactions. arXiv.

[120] Mallya A., Lazebnik S. (2016). Learning models for actions and person-object interactions with transfer to question answering. Proceedings of European Conference on Computer Vision.

[121] Gkioxari G., Girshick R., Dollár P., He K. (2017). Detecting and recognizing human-object interactions. arXiv.

[122] Gorban A., Idrees H., Jiang Y., Zamir A.R., Laptev I., Shah M., Sukthankar R. THUMOS challenge: Action recognition with a large number of classes. Proceedings of the CVPR Workshop.

[123] Shou Z., Wang D., Chang S.-F. Temporal action localization in untrimmed videos via multi-stage cnns. Proceedings of the IEEE Conference on Computer Vision and Pattern Recognition 2016.

[124] Yu G., Yuan J. Fast action proposals for human action detection and search. Proceedings of the IEEE Conference on Computer Vision and Pattern Recognition 2015.

[125] Weinzaepfel P., Harchaoui Z., Schmid C. Learning to track for spatio-temporal action localization. Proceedings of the IEEE International Conference on Computer Vision 2015.

[126] Heilbron F.C., Escorcia V., Ghanem B., Niebles J.C. ActivityNet: A large-scale video benchmark for human activity understanding. Proceedings of the IEEE Conference on Computer Vision and Pattern Recognition 2015.

[127] A Large-Scale Video Benchmark for Human Activity Understanding. http://activity-net.org/index.html.

